# Psychological safety and accountability in longitudinal integrated clerkships: a dual institution qualitative study

**DOI:** 10.1186/s12909-023-04622-5

**Published:** 2023-10-12

**Authors:** Robyn A. Latessa, Shelley L. Galvin, Robert A. Swendiman, Joshua Onyango, Bayla Ostrach, Amy C. Edmondson, Scott A. Davis, David A. Hirsh

**Affiliations:** 1 Department of Family Medicine, University of North Carolina School of Medicine, Chapel Hill, NC USA; 2 University of North Carolina Health Sciences at Mountain Area Health Education Center, Asheville, NC USA; 3 Department of Obstetrics and Gynecology, Mountain Area Health Education Center, Asheville, NC USA; 4 Department of Pediatric General Surgery, University of Utah School of Medicine, Salt Lake City, UT USA; 5 Department of Internal Medicine, Yale School of Medicine, New Haven, CT USA; 6 Departments of Family Medicine and Medical Anthropology, Boston University School of Medicine, Boston, MA USA; 7 Harvard Business School, Boston, MA USA; 8 Division of Pharmaceutical Outcomes and Policy, University of North Carolina Eshelman School of Pharmacy, Asheville, NC USA; 9 Harvard Medical School, Boston, MA USA; 10 Department of Medicine, Cambridge Health Alliance, 1493 Cambridge Street, Cambridge, MA 02139 USA

**Keywords:** Psychological safety, Accountability, Longitudinal integrated clerkships, Clinical education, Trust, Medical student-teacher relationship, Preceptor

## Abstract

**Background:**

Psychological safety and accountability are frameworks to describe relationships in the workplace. Psychological safety is a shared belief by members of a team that it is safe to take interpersonal risks. Accountability refers to being challenged and expected to meet expectations and goals. Psychological safety and accountability are supported by relational trust. Relational continuity is the educational construct underpinning longitudinal integrated clerkships. The workplace constructs of psychological safety and accountability may offer lenses to understand students’ educational experiences in longitudinal integrated clerkships.

**Methods:**

We performed a qualitative study of 9 years of longitudinal integrated clerkship graduates from two regionally diverse programs—at Harvard Medical School and the University of North Carolina School of Medicine. We used deductive content analysis to characterize psychological safety and accountability from semi-structured interviews of longitudinal integrated clerkship graduates.

**Results:**

Analysis of 20 graduates’ interview transcripts reached saturation. We identified 109 discrete excerpts describing psychological safety, accountability, or both. Excerpts with high psychological safety described trusting relationships and safe learning spaces. Low psychological safety included fear and frustration and perceptions of stressful learning environments. Excerpts characterizing high accountability involved increased learning and responsibility toward patients. Low accountability included students not feeling challenged. Graduates’ descriptions with both high psychological safety and high accountability characterized optimized learning and performance.

**Conclusions:**

This study used the workplace-based frameworks of psychological safety and accountability to explore qualitatively longitudinal integrated clerkship graduates’ experiences as students. Graduates described high and low psychological safety and accountability. Graduates’ descriptions of high psychological safety and accountability involved positive learning experiences and responsibility toward patients. The relational lenses of psychological safety and accountability may inform faculty development and future educational research in clinical medical education.

## Background

Longitudinal integrated clerkships (LICs) are educational structures in which medical students undertake their core clinical year engaging in patient care longitudinally across multiple medical disciplines simultaneously [1–3]. LICs provide students “educational continuity” [1] through longitudinal patient and preceptor relationships over periods of at least 6 months [2–7]. The literature reports LICs’ academic and professional outcomes: LICs compare favorably with traditional block clerkships (TBCs) in terms of students’ examination scores and clinical assessments, and LIC students report greater patient-centeredness and satisfaction [2, 3, 8–10]. Evidence reports preceptors’ satisfaction [11–14] and positive outcomes for patients and communities [4, 15–18]. Educational leaders and researchers are seeking further investigations to characterize elements of LICs that may support these outcomes [2, 4, 6, 7]. Recent research suggests that students’ longitudinal relationships with patients, preceptors, peers, and place offer important affordances for learning in the core clinical year [19]. *How* these relationships affect students within their clinical year remains less clear [19, 20]. The literature in K-12 education [21] and the business literature describing workplaces [22, 23] demonstrate how relationships characterized by high psychological safety (PS) and accountability form the foundation of learning, meaningful roles, and professional development.

**Fig. 1:**
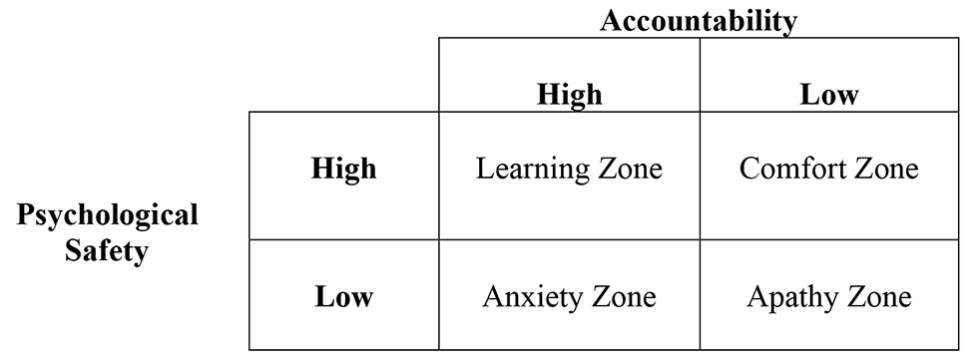
Edmondson’s Taxonomy of Archetypal Zones. Adapted from: Edmondson AC. *Teaming: How Organizations Learn, Innovate, and Compete in the Knowledge Economy*. John Wiley & Sons; 2012

Building on Schein and Bennis, Edmondson developed the frameworks of PS and accountability through descriptions and empirical studies of work environments [24, 25]. Edmondson at al. define PS as “the degree to which people view [their] environment as conducive to interpersonally risky behaviors like speaking up or asking for help” [24]. Studies of PS include wide ranging work environments. In healthcare, PS stands out as “especially important for enabling learning and change in contexts characterized by high stakes, complexity, and essential human interactions” [24]. Accountability is also critical for successful learning [26] and safe and effective workplaces [27]. Edmondson defines accountability as “the degree to which people are expected to adhere to high standards and pursue challenging goals” [25]. Because PS and accountability interrelate, Edmondson places them as axes of a 2 × 2 table creating four “Zones”: the Learning Zone (high PS, high accountability), Comfort Zone (high PS, low accountability), Anxiety Zone (low PS, high accountability), and Apathy Zone (low PS, low accountability) (Fig. 1) [25]. The Learning Zone is characteristic of high performing workplace teams [25].

Studies considering PS and accountability in the student-preceptor relationship in medical education remain nascent [28]. Two recent publications characterize PS in medical education. Thyness et al. performed a cross-sectional study of European medical students’ experiences of supervised patient encounters [29]. The authors found the strongest positive associations between PS and a supervisor’s clinical coaching and modeling behaviors [29]. McClintock et al. reported a dual-institution qualitative study of fourth-year medical students [30]. The authors describe themes that promote PS: “relationships, an emphasis on learning, clear expectations, autonomy, and frequent feedback” ([30], p. S46).

The literature in fields outside of medical education describes benefits of PS and accountability: effective teamwork [31]; effective team leadership and the quality and safety of complex processes [22, 25]; innovation and learning [26]; and professional development [24, 27]. Because these needs are critical to healthcare, medical education should also incorporate PS and accountability. Our review of the literature uncovered no studies exploring graduates’ experiences of PS and accountability during their core clinical year of medical school. Because Edmondson’s framework is a relational model [27] to explain performance and learning in workplace environments, PS and accountability may offer lenses through which to understand LICs. In this study, we qualitatively investigated LIC graduates’ reflections upon being students during their LIC year; we aimed to determine whether or how they experienced PS and accountability as they engaged longitudinally with their preceptors and patients. Our overarching goal was to characterize features of effective longitudinal clinical placements. The framework of PS and accountability may further characterize relational medical education and its effect on students and offer implications for faculty development.

## Methods

### Study design, participants, and setting

We performed a qualitative study of semi-structured interviews of students, residents, and attending physicians as they reflected back on their core clerkship year of medical school. Our choice to employ semi-structured interviews was based on the characteristics defined in the Robert Wood Johnson Foundation’s Qualitative Research Guidelines Project [32]. All participants were LIC program graduates (hereafter “graduates”) who had completed an LIC at one of two regionally diverse institutions: the Harvard Medical School Cambridge Integrated Clerkship (HMS-CIC) or the University of North Carolina (UNC) School of Medicine Longitudinal Integrated Clerkship in Asheville, North Carolina. The HMS-CIC is a 12-month LIC taking place at Cambridge Health Alliance, an academic, public safety net institution, and one of HMS’s core teaching hospitals. In this program, students complete all required disciplines of HMS’s core clinical year: internal medicine, surgery, obstetrics/gynecology, pediatrics, neurology, psychiatry, and radiology. UNC’s Asheville LIC program is a 12-month LIC across the same specialties as HMS-CIC plus family medicine. Students’ clinical experience occurs at Mission Hospitals, rural hospitals, Mountain Area Health Education Center clinics, and within physicians’ private practices, core teaching sites for UNC School of Medicine. These programs are described in further detail elsewhere [9, 33, 34]. The student participants completed their programs between 2004 and 2013. Recruitment of participants took place as part of another study reported previously [19]. The Institutional Review Boards of HMS, UNC, and Cambridge Health Alliance waived the need of ethical approval for this research protocol.

### Data collection

We electronically surveyed participants about their experiences in LIC programs. The survey was open to participants from November 11, 2013 to December 30, 2013. After completion of the survey, respondents were invited to participate in an optional interview. The interview guide consisted of four questions. We published a mixed methods analysis including answers to the first question (about affordances for learning opportunities that students perceive) and the fourth question (“Is there anything else you would like to add?”) in an earlier manuscript [19]. The first question asked, “What factors about your experiences in your third year longitudinal integrated curriculum do you think contributed most to your success?” This current study of PS and accountability has a different focus and uses a different method to analyze responses to questions one, two, three, and four. Question two is “Tell me about one of your best learning experiences during the third year and what made it so great?” Question three is “What was your worst experience and why?” Question four asks, “Is there anything else you would like to add?”

At the outset, we enrolled a convenience sample of the first 10 eligible volunteers from each LIC program to participate in telephone interviews (n = 20). We did not collect demographic information on the anonymous volunteers. We were prepared to recruit additional volunteers in each program until the deductive qualitative research process reached a priori thematic saturation: “the degree to which identified codes or themes are exemplified in the data” [35]. A trained research assistant with no involvement with any LIC program conducted and recorded semi-structured interviews using the aforementioned four questions. The research assistant was overseen and involved in iterative training in interviewing by an established professional mixed methods researcher (SLG) with 25 years of experience. The research assistant did several practice interviews that were critiqued and debriefed by SLG to refine the processes before commencing the interviews. There were no explicit probes included in the interview guide, but the interviewer was trained to ask probing questions to elicit details from respondents. The length of the interviews varied between 14 and 40 min (median of 25.5 min). All 20 interviews were transcribed and de-identified between December 5, 2013 and March 11, 2014.

### Data analysis

From September 2016 to March 2017, we analyzed the interview transcripts using deductive content analysis [36]. This qualitative approach is employed to investigate an existing theory or conceptual framework in a new context [36], as was the nature of our study of PS and accountability in clinical education.

We began by summarizing relevant research findings about PS and accountability to guide initial codes [37, 38]. Two investigators (SLG and JO) independently coded three interview transcripts to probe for the themes of PS and accountability. SLG is an academic researcher at UNC with experience in qualitative research and no connection to LIC programs. JO was then a medical student in UNC’s LIC who received basic training in qualitative research for this study. These investigators met serially, compared their coding, revisited the literature for construct clarification, resolved discrepancies through discussion, and revised codes as necessary. SLG and JO then joined another investigator (RAL), who directs the UNC LIC, to review and clarify codes. RAL is an academic researcher with experience in qualitative research.

SLG and JO then independently coded five more interview transcripts and met to discuss and reach consensus. Subsequently, the two researchers discussed and clarified the coding of these additional five transcripts with RAL. SLG. and JO then independently finished coding all remaining interview transcripts. JO and SLG compared their coding of these ten transcripts and reconciled differences to reach consensus.

As part of the coding, to determine the nature of descriptions of PS and accountability, we identified subsections of interviewees’ responses to a question. We refer to these elements as “excerpts” and delineated each excerpt when an interviewee’s idea had a natural break before another idea began (i.e., a switch from describing one interaction or one preceptor to different example). We included excerpts describing PS and accountability when the narrative was based on interviewees’ descriptions of a personal clinical experience involving a patient, preceptor, or both and excluded responses describing perceptions of other people’s experiences.

We took approaches to support trustworthiness [39, 40]. Regarding credibility, researchers immersed themselves in the content of interview transcripts, kept reflective notes, considered reflexivity serially, undertook peer debriefing, and queried the data counterfactually (seeking other explanations for findings). We ensured our process reached a priori thematic saturation [35] with the interview data. We performed member checking. Regarding dependability, we determined that data remained stable over time using logs, reflection, and debriefing meetings. We describe the status of participants and describe the educational context to offer considerations for transferability. Regarding confirmability, we upheld standard audit trails of notes and processes and undertook recurring peer reviews. For conformability, we provide illustrative quotes from a range of participants about core concepts, after serial review by all researchers.

## Results

Interviewees included 20 participants: 10 graduates of HMS’s LIC and 10 graduates of UNC’s LIC. We identified 109 discrete excerpts. Below, we present results of our deductive content analysis. Excerpts include graduates’ descriptions of behaviors or conditions associated with PS (high or low PS), with accountability (high or low accountability), or both PS and accountability.

### Psychological safety

#### High psychological safety

Interviewees described behaviors essential to PS, such as engagement, reflection, and asking for help. These excerpts included descriptions of high-quality relationships that developed over time and enhanced trust in the student. Interviewees suggested that supportive longitudinal relationships created a context for learning from challenge and taking learning risks without fear of consequences.

*I felt that my preceptors really got to know me as a person and a student and so were better able to set goals for me and push me to be successful. I think that was really important for me. I think I also felt more comfortable with them and felt more that it was safer to make mistakes. And I think that helped me kind of work or push myself beyond my comfort zone and…make a plan for a patient or…say what I thought was going on even if I wasn’t sure I was right.* [Interviewee #1]

Interviewees focused on preceptors arranging safe opportunities for medical students to practice medicine within the scope of their training. Interviewees described experiences that involved preceptor-facilitated PS and focused on longitudinal relationships with preceptors. Interviewees suggested that supportive longitudinal relationships created possibilities for increased independence which generated learning benefits.

*I would say there were some preceptors with whom my relationship grew throughout the year, and that really allowed for some tremendous education. I think about my medicine preceptor who…treated me with a high degree of autonomy and intellectual respect that…allowed me to be challenged.* [Interviewee #14]

#### Low psychological safety

Excerpts that encapsulated low PS described interactions that felt personal rather than focused on educational topics. These longitudinal relationships featured communication that prompted students’ fearfulness in asking for help, sharing ideas, or experimenting. Some interviewees described their relationships getting off to a bad start or described times when they were not given the benefit of the doubt.

*I had a difficult time with…a significant personality clash with a preceptor, and I had difficulty navigating it. It was early in my third year… I had a difficult time learning from that person because the focus seemed to be on me and what I was good or not good at as opposed to the material and learning and growth. So it was…‘you do this well and this not well’, as opposed to teaching.* [Interviewee #8]

Some interviewees reported interpersonal struggles with other members of the healthcare team and difficult communication related to hierarchy—stressors related to low PS in workplaces. In commenting on their clinical encounters, interviewees distinguished challenges that built resilience and challenges that undermined resilience; whereas productive challenge could be energizing, distressing challenge impeded learning. The longitudinal structure afforded the benefit of time to resolve relational issues or created difficulty when disempowered students felt unable to change circumstances with low PS. Interviewees’ excerpts that suggested low PS in these longitudinal clinical workplaces, described frustration, fear, or distress.

*And everything I did…was wrong…There was one time when the nurses were chatting with me…and she cut me off and said, “You are not good enough to talk while you are doing something!” And so that was challenging because it felt like I couldn’t do anything right. There is a point that tough love makes you work harder to know the answers to the tough questions, and there is a point where it is pointless, and so no matter what you do you are going to be wrong and that is just discouraging. And I don’t think I learned very much because you get so frustrated that it is hard to glean anything from the experience.* [Interviewee #17]

### Accountability

#### High accountability

Interviewees characterized high accountability in terms of tailored learning experiences with high expectations. Some interviewees named the importance of recurring assignments/readings to be completed over time. Other interviewees described the importance of longitudinal clinical oversight and iterative feedback to increase accountability over time. The LIC structure, which creates possibilities for students to see patients across venues of care, supported students’ sense of accountability. Interviewees described how over the longitudinal year, they were continually accountable to their preceptor which translated into being accountable to their patients. They described a sense of duty and commitment and connected a sense of duty to enhancing learning and responsibility.

*[For] each patient, I would have a learning issue that I would be accountable for the next week. I saw those patients in succession during the course of the year, and I followed several into the hospital and into referral appointments. And that was helpful in having a sense of ownership…and accountability to the patient. That was more learning–that taught me more about how to be a doctor for a patient–the person who is in charge of someone’s healthcare.* [Interviewee #18]

Reflecting on their longitudinal clinical experiences, interviewees also connected accountability, agency, and independence. In addition to the aforementioned affordances arising from an increased sense of duty and commitment, independence allowed opportunities for learning and to enhance the student’s role in patients’ care. Interviewees referred to longitudinal care and learning and also to longitudinal relationships with preceptors. Accountability could arise from their longitudinal teacher-student interactions. Sometimes when a longitudinal preceptor was perceived as a hard preceptor, interviewees mentioned accountability in negative terms and other times in positive terms; learning, independence, and role responsibility over time appeared connected to positive framings.

*In my medicine clinic, I worked with a very notoriously intense preceptor. In a good way, he gave students a lot of responsibility and independence and that fit well with my learning style…I got to feel like the actual primary care provider for these patients. I had the appropriate support from my preceptor but at the same time I had enough space…[to] learn a ton.* [Interviewee #4]

#### Low accountability

Longitudinal design of education could also be frustrating if interviewees were not held to high standards. Interviewees described the value of being appropriately challenged and that learning could be undermined if they were not sufficiently challenged by their yearlong preceptors. Interviewees noted that positive longitudinal relationships needed to include adequate accountability to ensure that learning was robust. Low accountability can be demotivating; these interviewees described circumstances that led to students’ disengagement and diminished effort. They reported “missed opportunities” for learning, as they sought more responsibility over time.

*I would say that sometimes throughout the year, I felt that my preceptors were too kind, too nice almost. I did feel that sometimes the preceptors let me off a little too easily, you know, didn’t push me hard enough to make sure that I really understood exactly all of my diagnoses.* [Interviewee #7]

### Psychological safety and accountability

Excerpts containing both PS and accountability could be placed in Edmondson’s four zones: the Learning Zone (high PS, high accountability), Comfort Zone (high PS, low accountability), Anxiety Zone (low PS, high accountability), and Apathy Zone (low PS, low accountability) (Fig. 1) [25]. We analyze transcripts that fit into each zone and provide one representative quotation from each zone below:

#### Learning zone

Interviewees in the Learning Zone emphasized trust that developed longitudinally over the course of the year working with particular clinical preceptors. Interviewees reported that when trust developed, longitudinal clinical teachers developed a willingness to grant responsibility to the student, an affordance for rapid learning and growth in their emerging role.

*My best learning experience was…[resident saying], ‘Today you have been upgraded. You are going to be helping me out as if you were a resident.’ Which of course was terrifying at that moment, but I think it was the most incredible experience I ever had…she really trusted me. It helped that I had worked with her multiple times over the course of the year. She trusted me and knew that I did a good job… Having that responsibility very quickly—they gave me that responsibility, and I jumped right into it. They supported me if I felt overwhelmed.* [Interviewee #6]

#### Comfort zone

Interviewees in the Comfort Zone emphasized barriers arising from low accountability. Low accountability included passive learning without interactive engagement or challenge. Interviewees repeatedly mentioned that they appreciate support, but they distinguish the value of support from the value of challenge. Interviewees sought challenge which they recognized was helpful to their learning. Interviewees found that low accountability undermined learning and was inefficient.

*On the first day, we saw a patient and then sat down, and he said, “Well what do you think you need to learn about?” And I said, “Well my last person had [a certain diagnosis], and I think it would be helpful to learn some about that.” He proceeded to talk for an hour and a half straight… He was a very nice guy, and he took so much time so I appreciate that so much, but I couldn’t learn by sitting in front of him for an hour and a half while he talked. I would have benefited from just reading or him asking me questions or going through things together rather than that.* [Interviewee #18]

#### Anxiety zone

Interviewees in the Anxiety Zone emphasized that the preceptor challenged them, but did not make them feel safe or comfortable to make mistakes. This feeling of reduced PS was more difficult given the longitudinal structure wherein students would be attending clinics recurrently for most of the year with their preceptor. Looking back, interviewees reflected that despite their negative emotional responses to the preceptor, they recognized legitimate challenges accrued from those experiences. It was not always clear how decreased PS coexisting with legitimate challenge ultimately affected longitudinal learning.

*My preceptor was just really tough on me…we went a whole year, and she didn’t even realize I was engaged, and I got engaged like the week before I started my rotation… Every single week I really dreaded going to that rotation and having to work under her. But I also think that in the back of my mind the thing that I dreaded the most about it was probably the fact that she did call me out on things I needed to work on…looking back it probably was not a negative experience but gosh she made that 10 months kind of go by pretty slowly…* [Interviewee #11].

#### Apathy zone

Interviewees in the Apathy Zone emphasized that they developed neither the trust nor the challenge necessary for learning and growth. This combination of low PS and low accountability could affect students’ experience such that they had less independence and could negatively affect their interest. Although interviewee transcripts rarely reported this circumstance, these events prompted program intervention, including providing students different longitudinal preceptors.

*I initially worked with a preceptor who I really didn’t click with. I was not given much autonomy… I wasn’t feeling challenged or interested, so I ended up moving to another clinic.* [Interviewee #14]

## Discussion

In this study at two medical schools, we used the lenses of PS and accountability to explore LIC graduates’ reflections on their experiences as students in LICs. Our qualitative analysis suggests that graduates perceived both high and low PS and high and low accountability in their clinical work with preceptors. This study appears to be the first to describe the interplay of PS and accountability in the core clinical year of medical school. This study builds upon literature describing affordances of trust, autonomy, and engagement [10, 12, 19, 41, 42] by characterizing how students perceived PS and accountability in their learning and their longitudinal student-preceptor relationships. Our findings suggest it is important for LIC faculty—and perhaps preceptors in other clinical education models—to be trained in fostering PS and accountability.

Our data, derived in the context of clinical medical education, align with studies that investigated PS in workplace settings and schooling outside of medical education. In our data and in other contexts, a key cofactor in PS appears to be trust. In business literature, high-quality relationships, grounded in trust, underpin psychologically safe work environments [22, 27, 43]. These trusting relationships are shown to support learning and effectiveness in workplaces and organizations [23, 27]. Trust has also been described as an essential element for relationships, institutional effectiveness, and learning within primary schools [21]. The medical education literature posits that trust supports medical students’ learning and caregiving [10, 28, 44] and residents’ satisfaction [45]. PS may also support core learning behaviors in medicine including experimentation, speaking up, collaboration, and reflection [25, 46, 47]—important components of professionalism [48], trauma-informed learning environments [49], and high quality, safe care [50, 51].

Our graduates characterized accountability to their preceptors and, at times, both PS and accountability within these relationships. Graduates reporting high PS and high accountability (i.e., the “Learning Zone”) described collaborative relationships with preceptors which enabled meaningful feedback and fostered graded responsibility. These findings within clinical education also build upon studies in workplace environments and primary/secondary schools. In workplaces, when high PS exists in a relationship, the experience of accountability may be motivating and engaging, rather than depleting or paralyzing [52]. In clinical education, high PS and high accountability facilitate teachers and students to develop open dialogue, shared goals, and mutual respect [53]. In workplace and scholastic environments, high PS and accountability allow supervisors to challenge learners and provide them opportunities to address mistakes and improve performance [22, 26]. In these contexts, PS may function to reduce power imbalances, and the supervisory person may be perceived by the supervisee as an ally [10, 47, 52], furthering accountability [54] and learning from “productive failure” [55, 56]. Considering studies from the workplace and scholastic literatures, our data may shed light on the LIC literature; PS and accountability may partly explain LIC students’ sense of responsibility for their patients over time [8] and their greater engagement than TBC peers in performing patient care independently and developing meaningful roles [11, 57].

Not all excerpts in our data describe high PS or high accountability. Graduates characterizing low PS described distress and fear of taking risks. Graduates characterizing low accountability described their sense of inadequate challenge, disengagement, and missed opportunities for learning. These findings align with studies from business and primary/secondary schools. Organizational research reports the importance of risk-taking for learning and performance and the importance of “learning from failures,” particularly in institutions engaged in healthcare delivery [22]. In scholastic environments, studies of risk-taking and its effect on learning suggest that PS and accountability may work together to improve performance, and if either PS or accountability is low, the result is decreased performance [26]. A longitudinal study of 58,000 K-12 teachers, in approximately 545 schools, demonstrated that “psychological safety acts as an accelerant, augmenting the powerful impact that high felt accountability can have on ameliorating performance over time [26].” Given the risk of decreased performance arising from low PS and/or low accountability, we believe this framework creates opportunities for faculty development.

Faculty development for LICs [58, 59], longitudinal courses [7, 10, 14], or shorter educational interactions, could incorporate the rationale for and means to support PS [60] and accountability, including training around Edmondson’s 2 × 2 construct (Fig. 1) [25]. Consistent with the Learning Zone, prior research suggests that LIC students value psychologically safe relationships with preceptors *and* value preceptors who set high expectations and standards of responsibility [59]. Our data in concert with business and scholastic literature suggest the role of “preceptor” contains supervisory and educational responsibilities: ideally, trusted overseer, trusted teacher, trusted collaborator. This characterization acknowledges the interplay of the workplace and educational duties of the preceptor and learner in clinical education and highlights the need to train faculty explicitly in the importance and methods of trust-building [28, 61, 62]. Faculty should learn how trust-building underpins PS and accountability and advances performance, learning, and caring [27, 63]. Training should include techniques to provide an educational alliance [64], effective, psychologically safe feedback [53], emotionally intelligent, inclusive oversight [51, 62], and autonomy supportive teaching (i.e. to support intrinsic motivation) [65].

Faculty development should also train preceptors to orient learners to their context, discuss standards at the outset of their relationship, invite learner perspectives, and practice ways to maintain PS and accountability especially in times of challenge for the preceptor or learner. If the preceptors and learners are afforded educational continuity [1], preceptors can increase accountability in an appropriately graduated fashion over time—collaborating to effectively meet the learner’s evolving educational needs [28, 61]. Faculty can also learn techniques to create high PS and foster accountability in brief interactions [26]. The PS study by McClintock et al. included mostly students who undertook traditional block rotations; consistent with our findings, the authors emphasize the importance of relationships in creating PS [30]. Although we did not study structures other than LICs, our results also highlight the connection between relationships and PS suggesting that regardless of model, relationships matter.

Training should also include approaches to identify and attend to learners who experience less PS and/or accountability. For example, although our graduates’ interviews preceded the coronavirus pandemic, we recognize the importance of PS in the context of student learning during this era. The strain of the COVID pandemic, societal tumult, and other stressors generate increased uncertainty and pressure on students and preceptors. Preceptors and program leaders should learn how to create environments with PS and accountability during such times [49, 66, 67].

In this study, graduates’ descriptions principally involved experiences with their preceptors. The concepts of PS and accountability might also be applied in the context of student-patient relationships, peer-peer relationships, and relationships between learners and communities they serve [1, 19]. Applications of PS and accountability beyond the student-preceptor dyad are areas for future study.

### Limitations

This research has limitations. This is a two-school study which limits generalizability. Although our analysis reached a priori thematic saturation, this study included the first 10 participants from each site who expressed interest, which may lead to selection bias. Including only 10 participants across nine years could miss richness of the experiences of others who were not interviewed. The interview portion of the study was a subset of the larger survey sample [19]; we did not collect interviewee demographics separately. We examined past perceptions of PS and accountability among LIC graduates, possibly introducing recall bias. Also, interviewees who graduated more recently would have fresher memories of their LIC experience; potentially, interviewees whose experiences were a long time previously could be recalling experiences that actually occurred after their LICs. We did not evaluate the perspectives of current students, TBC students, other institutions’ LIC graduates, preceptors, or patients and did not triangulate our data. The dichotomization of best and worst educational experiences during the interviews may encourage extreme examples of high and low PS and accountability and may not represent the actual range of experiences among interviewees.

## Conclusions

This study investigated PS and accountability in longitudinal relationships during the clinical year of medical school. LIC graduates described their experiences as students working with preceptors over time, and their descriptions depicted PS and accountability. This study in a medical education context aligns with findings from the business and scholastic literatures. PS and accountability offer medical education a framework for faculty development and for research into relationships among preceptors, patients, and medical students. Ultimately, PS and accountability may further the goals of creating and maintaining positive and effective educational relationships.

## Data Availability

The interview data generated and analyzed during this study are not publicly available as our Institutional Review Board submission and determination did not state explicitly that data would be publicly available. Within the limits of Institutional Review Board and standard considerations, data may potentially be available upon reasonable request directed to the corresponding author.

## List of abbreviations

PS: Psychological Safety
LIC: Longitudinal Integrated Clerkship
TBC: Traditional Block Clerkship
HMS: Harvard Medical School
HMS-CIC: Harvard Medical School Cambridge Integrated Clerkship
UNC: University of North Carolina
COVID: Coronavirus disease

## References

[1] Hirsh DA, Ogur B, Thibault GE, Cox M (2007). Continuity as an organizing principle for clinical education reform. N Engl J Med.

[2] Alliance for Clinical Education (2016). Longitudinal integrated clerkships: Principles, outcomes, practical tools, and future directions..

[3] Hirsh D, Singh TA, Saravanan Y, Walters LK. Learning in longitudinal integrated clerkships. In: Dent JA, Harden RM, Hunt D, editors. A practical guide for Medical Teachers. 6th ed. Elsevier Health Sciences; 2021. pp. 84–91.

[4] Walters L, Greenhill J, Richards J, Ward H, Campbell N, Ash J (2012). Outcomes of longitudinal integrated clinical placements for students, clinicians and society. Med Educ.

[5] Worley P, Couper I, Strasser R, Graves L, Cummings BA, Woodman R (2016). A typology of longitudinal integrated clerkships. Med Educ.

[6] Ellaway RH, Graves L, Cummings BA (2016). Dimensions of integration, continuity and longitudinality in clinical clerkships. Med Educ.

[7] Gheihman G, Jun T, Young GJ, Liebman D, Sharma K, Brandes E (2018). A review of longitudinal clinical programs in US medical schools. Med Educ Online.

[8] Ogur B, Hirsh D (2009). Learning through longitudinal patient care-narratives from the Harvard Medical School-Cambridge Integrated Clerkship. Acad Med.

[9] Hirsh D, Gaufberg E, Ogur B, Cohen P, Krupat E, Cox M (2012). Educational outcomes of the Harvard Medical School-Cambridge integrated clerkship: a way forward for medical education. Acad Med.

[10] Hauer KE, Hirsh D, Ma I, Hansen L, Ogur B, Poncelet AN (2012). The role of role: learning in longitudinal integrated and traditional block clerkships. Med Educ.

[11] O’Brien BC, Poncelet AN, Hansen L, Hirsh DA, Ogur B, Alexander EK (2012). Students’ workplace learning in two clerkship models: a multi-site observational study. Med Educ.

[12] Hauer KE, O’Brien BC, Hansen LA, Hirsh D, Ma IH, Ogur B (2012). More is better: students describe successful and unsuccessful experiences with teachers differently in brief and longitudinal relationships. Acad Med.

[13] Snow SC, Gong J, Adams JE (2017). Faculty experience and engagement in a longitudinal integrated clerkship. Med Teach.

[14] Shagrin BS, Gheihman G, Sullivan AM, Li H, Hirsh DA. Faculty perspectives on facilitating medical students’ longitudinal learning: A mixed-methods study. Med Educ. 2022. 10.1111/medu.14842 35599241

[15] Flick RJ, Felder-Heim C, Gong J, Corral J, Kalata K, Marin A (2019). Alliance, Trust, and loss: experiences of patients cared for by students in a Longitudinal Integrated Clerkship. Acad Med.

[16] Konkin J, Suddards C (2012). Creating stories to live by: caring and professional identity formation in a longitudinal integrated clerkship. Adv Health Sci Educ Theory Pract.

[17] Hirsh D, Walters L, Poncelet AN (2012). Better learning, better doctors, better delivery system: possibilities from a case study of longitudinal integrated clerkships. Med Teach.

[18] Chang YW, Hirsh DA, Fang WH, Li H, Tzeng WC, Kao S (2021). Patient perceptions of students in a longitudinal integrated clerkship in Taiwan: a qualitative study. BMC Med Educ.

[19] Latessa RA, Swendiman RA, Parlier AB, Galvin SL, Hirsh DA (2017). Graduates’ perceptions of learning Affordances in Longitudinal Integrated Clerkships: a Dual-Institution, mixed-methods study. Acad Med.

[20] Bates J, Konkin J, Suddards C, Dobson S, Pratt D (2013). Student perceptions of assessment and feedback in longitudinal integrated clerkships. Med Educ.

[21] Bryk AS, Schneider B (2003). Trust in schools: a core resource for school reform. Educ Leadersh.

[22] Carmeli A, Gittell JH (2009). High-quality relationships, psychological safety, and learning from failures in work organizations. J Organ Behav.

[23] Clark TR. The 4 stages of Psychological Safety: defining the path to inclusion and Innovation. Berrett-Koehler Publishers; 2020.

[24] Edmondson AC, Higgins M, Singer S, Weiner J (2016). Understanding psychological safety in health care and education organizations: a comparative perspective. Res Hum Dev.

[25] Edmondson AC. Teaming: how Organizations learn, innovate, and compete in the Knowledge Economy. John Wiley & Sons; 2012.

[26] Higgins M, Weiner J (2014). Weathering the storm: Effects of psychological safety and accountability on performance. Acad Manag Proc.

[27] Edmondson AC. Psychological safety, trust, and learning in organizations: a group-level lens. In: Kramer RM, Cook KS, editors. Trust and Distrust in Organizations: dilemmas and approaches. Russell Sage Foundation; 2004. pp. 239–72.

[28] Hauer KE, Ten Cate O, Boscardin C, Irby DM, Iobst W, O’Sullivan PS (2014). Understanding trust as an essential element of trainee supervision and learning in the workplace. Adv Health Sci Educ Theory Pract.

[29] Thyness C, Grimstad H, Steinsbekk A (2023). Psychological safety in european medical students’ last supervised patient encounter: a cross-sectional survey. PLoS ONE.

[30] McClintock AH, Fainstad TL, Jauregui J (2022). Clinician teacher as leader: creating Psychological Safety in the clinical learning environment for medical students. Acad Med.

[31] Appelbaum NP, Lockeman KS, Orr S, Huff TA, Hogan CJ, Queen BA (2020). Perceived influence of power distance, psychological safety, and team cohesion on team effectiveness. J Interprof Care.

[32] Cohen D, Crabtree B. Qualitative research guidelines project. 2006. https://sswm.info/sites/default/files/reference_attachments/COHEN%202006%20Semistructured%20Interview.pdf. Accessed 17 February 2023.

[33] Ogur B, Hirsh D, Krupat E, Bor D (2007). The Harvard Medical School-Cambridge integrated clerkship: an innovative model of clinical education. Acad Med.

[34] Latessa R, Beaty N, Royal K, Colvin G, Pathman DE, Heck J (2015). Academic outcomes of a community-based longitudinal integrated clerkships program. Med Teach.

[35] Saunders B, Sim J, Kingstone T, Baker S, Waterfield J, Bartlam B (2018). Saturation in qualitative research: exploring its conceptualization and operationalization. Qual Quant.

[36] Elo S, Kyngäs H (2008). The qualitative content analysis process. J Adv Nurs.

[37] Hsieh HF, Shannon SE (2005). Three approaches to qualitative content analysis. Qual Health Res.

[38] Tong A, Sainsbury P, Craig J (2007). Consolidated criteria for reporting qualitative research (COREQ): a 32-item checklist for interviews and focus groups. Int J Qual Health Care.

[39] Elo S, Kaariainen M, Kanste O, Polkki T, Utriainen K, Kyngäs H (2014). Qualitative content analysis: a focus on trustworthiness. SAGE open.

[40] Connelly LM (2016). Trustworthiness in qualitative research. Medsurg Nurs.

[41] Teherani A, Irby DM, Loeser H (2013). Outcomes of different clerkship models: longitudinal integrated, hybrid, and block. Acad Med.

[42] Wamsley MA, Dubowitz N, Kohli P, Cooke M, O’Brien BC (2009). Continuity in a longitudinal out-patient attachment for Year 3 medical students. Med Educ.

[43] Carmeli A (2007). Social capital, psychological safety and learning behaviours from failure in organisations. Long Range Plann.

[44] Konkin DJ, Suddards C (2017). Students’ experiences of role, relationships and learning in two clerkship models. Med Educ.

[45] Torralba KD, Loo LK, Byrne JM, Baz S, Cannon GW, Keitz SA (2016). Does Psychological Safety Impact the Clinical Learning Environment for Resident Physicians? Results from the VA’s Learners’ perceptions Survey. J Grad Med Educ.

[46] Edmondson AC (2003). Speaking up in the operating room: how team leaders promote learning in interdisciplinary action teams. J Manag Stud.

[47] Gaufberg E, Bor D, Dinardo P, Krupat E, Pine E, Ogur B (2017). In pursuit of Educational Integrity: professional identity formation in the Harvard Medical School Cambridge Integrated Clerkship. Perspect Biol Med.

[48] Royal College of Physicians and Surgeons of Canada. CanMEDS: Better standards, better physicians, better care 2022 [Available from: royalcollege.ca/rcsite/canmeds/canmeds-framework-e.

[49] Brown T, Berman S, McDaniel K, Radford C, Mehta P, Potter J (2021). Trauma-informed Medical Education (TIME): advancing curricular content and Educational Context. Acad Med.

[50] Leape LL, Shore MF, Dienstag JL, Mayer RJ, Edgman-Levitan S, Meyer GS (2012). Perspective: a culture of respect, part 2: creating a culture of respect. Acad Med.

[51] Appelbaum NP, Dow A, Mazmanian PE, Jundt DK, Appelbaum EN (2016). The effects of power, leadership and psychological safety on resident event reporting. Med Educ.

[52] Nembhard IM, Edmondson AC (2006). Making it safe: the effects of leader inclusiveness and professional status on psychological safety and improvement efforts in health care teams. J Organ Behav.

[53] Johnson CE, Keating JL, Molloy EK (2020). Psychological safety in feedback: what does it look like and how can educators work with learners to foster it?. Med Educ.

[54] Romme AG (2019). Climbing up and down the hierarchy of accountability: implications for organization design. J Organ Design.

[55] Steenhof N, Woods NN, Mylopoulos M (2020). Exploring why we learn from productive failure: insights from the cognitive and learning sciences. Adv Health Sci Educ Theory Pract.

[56] Steenhof N, Woods NN, Van Gerven PWM, Mylopoulos M (2019). Productive failure as an instructional approach to promote future learning. Adv Health Sci Educ Theory Pract.

[57] O’Brien BC, Hirsh D, Krupat E, Batt J, Hansen LA, Poncelet AN (2016). Learners, performers, caregivers, and team players: descriptions of the ideal medical student in longitudinal integrated and block clerkships. Med Teach.

[58] Bernstein J, Wood S, Latessa R, Hirsh DA (2019). Teaching in longitudinal integrated clerkships: the seven ‘C’s. Clin Teach.

[59] Latessa R, Schmitt A, Beaty N, Buie S, Ray L (2016). Preceptor teaching tips in longitudinal clerkships. Clin Teach.

[60] Cave D, Pearson H, Whitehead P, Rahim-Jamal S (2016). CENTRE: creating psychological safety in groups. Clin Teach.

[61] Hirsh DA, Holmboe ES, ten Cate O (2014). Time to trust: longitudinal integrated clerkships and entrustable professional activities. Acad Med.

[62] George JM (2000). Emotions and leadership: the role of emotional intelligence. Hum Relations.

[63] Balmer DF, Hirsh DA, Monie D, Weil H, Richards BF (2016). Caring to Care: applying Noddings’ Philosophy to Medical Education. Acad Med.

[64] Telio S, Regehr G, Ajjawi R (2016). Feedback and the educational alliance: examining credibility judgements and their consequences. Med Educ.

[65] Reeve J, Cheon SH (2021). Autonomy-supportive teaching: its malleability, benefits, and potential to improve educational practice. Educ Psychol.

[66] Anderson N, Boatright D, Reisman A (2020). Blackface in White Space: using admissions to address racism in Medical Education. J Gen Intern Med.

[67] Kemp MT, Rivard SJ, Anderson S, Audu CO, Barrett M, Fry BT (2021). Trainee Wellness and Safety in the Context of COVID-19: the experience of one Institution. Acad Med.

